# Decomposing socioeconomic inequalities in alcohol use by men living in South African urban informal settlements

**DOI:** 10.1186/s12889-018-5925-4

**Published:** 2018-08-09

**Authors:** Nozuko Lawana, Frederik Booysen

**Affiliations:** 1 Population Health, Health Systems and Innovation, Human Sciences Research Council , 134 Pretorious Street, Pretoria, 0002 South Africa; 2 Department of Economics, University of Free State (UFS), Bloemfontein, South Africa

**Keywords:** Alcohol use, Socioeconomic inequalities, Concentration index, Decomposition, Informal settlements, South Africa

## Abstract

**Background:**

The prevalence of alcohol consumption among males living in urban settlements in South Africa is high. This paper aims to measure socioeconomic inequalities in alcohol use among men residing in informal settlements and also to examine the factors associated with inequality in alcohol use among men living in informal settlements.

**Methods:**

The study uses data from the 2016 Study of South African Informal Settlements. Multiple correspondence analysis is used to calculate a wealth index as a measure of socioeconomic status. The Erreygers concentration index is employed to quantify the degree of socioeconomic inequality in alcohol use and decomposition analysis is conducted to assess the factors associated with inequality in alcohol use by men of various age groups.

**Results:**

There is a socioeconomic-related inequality in alcohol use in informal settlements that discriminates against poor men. Inequality is especially pronounced in the case of males aged 15–34 years and males aged 35–44 years. Wealth status makes the biggest contribution to socioeconomic inequality in alcohol use. The contribution of social determinants of health like marital status and employment status differ across age groups. Employment status contribute more to the alcohol use inequality among males aged 15–34 years while marital status contribute more to the alcohol use inequality among males aged 35–44 years. Being single substantially increases inequality in alcohol use.

**Conclusion:**

Inequality in alcohol use exists among both younger and older males and discriminate against the poor. Public policies aimed at promoting public health and the prevention of unhealthy behaviours should target younger and middle-aged men from socioeconomically disadvantaged groups. We also suggest policies that target single males in informal settlements.

## Background

Individuals living in informal settlement are more likely to experience disease, injuries and premature death, and ill health [1]. Residents of informal settlements also have more limited access to health care services, often lives in overcrowded housing, and faces food insecurity on a daily basis. UN Habitat [2] argues that living in informal settlements often poses significant social problems, for example, access to services may be limited and overcrowding can contribute to stress, violence and increased problems of alcohol dependence. There are also many illegal liquor vendors in informal settlements. The number of informal liquor retailers or shebeens in South Africa has been estimated at between 190,000 and 265,000, with the majority located in informal settlements [3].

Heavy alcohol consumption is a well-known risk factor that is commonly associated with reduced production, increased injuries, sexual transmitted diseases, HIV/AIDS infections, exacerbated violence and more prevalent chronic diseases [4–6]. The 2010 global burden of diseases study describes alcohol use as the top risk factor accounting for the quadruple burden of diseases in South Africa. Alcohol misuse remains a serious problem among South Africans, as almost a third of heads of households in informal urban areas who participated in SANHANES-I regarded alcohol misuse as a serious or very serious problem [7].

Despite the evidence showing the negative aspects of alcohol use, the prevalence of drinking is still high in South Africa. It is estimated that about 33.1% of the South African population drink alcohol [8]. Males (33.7–47.7%) are also found more likely to report alcohol use than females (9.9–20.20%) [8–10]. Nguyen et al. [11], found that alcohol use is common in males as well as young adults. Urban South African residents, especially those living in informal urban settlements, were found to be more likely than rural residents to report current alcohol use [9, 10].

Previous studies have shown that inequalities in alcohol use is associated with socioeconomic status [4, 12, 13]. Ascertaining the extent of inequalities in alcohol use is crucial since there are different socioeconomic classes living within informal settlements and some residents are poorer than others. An understanding of the factors that are contributing to inequalities in alcohol use in informal settlements can help to inform and target effective and appropriate interventions. Factors like education and income have been identified as major contributing factors to inequality of alcohol use. In a recent article, Bockerman et al. [14] found a significant negative relationship between alcohol consumption and labour market outcomes using OLS regressions. Heavy drinkers and abstainers have substantially weaker labour market attachment (earnings and employment months) compared to moderate drinkers.

The primary objective of this paper is to decompose the socioeconomic inequalities in alcohol use by men living in informal settlements in South Africa. The specific research questions are: in which socioeconomic group is alcohol use more prevalent in South African informal settlements? What is the extent and nature of socioeconomic inequalities in alcohol use? What factors predict socioeconomic inequality in alcohol use among men living in informal settlements? This paper contributes to the literature by providing new evidence about the socioeconomic inequality in alcohol use among men living in informal settlements in South Africa. Moreover, a number of previous studies had decomposed the determinants of socioeconomic gradient in alcohol use in developed countries, with none done for developing countries, especially focusing on one of the most vulnerable segment of the country’s population. To our knowledge, this is the first study that decompose inequality in alcohol use of men living in informal settlements.

## Methods

### Data

The data source is the nationally representative Study of South African Informal Settlements conducted in 2015/2016. The survey received a clearance from the Research Ethics Committee (REC) of the HSRC (REC 9/21/05/14). Respondents were interviewed on a range of questions, including their type of dwelling structure, socioeconomic status, demographics, and lifestyle risk factors. The dataset used in this paper consist of socioeconomic data (type of dwelling and household assets) extracted from the household survey and data on lifestyle risk factors and demographics extracted from the individual questionnaire. The survey consisted of 3330 households, 3088 were valid, occupied household, of which 93% agreed to participate in the survey. This resulted in 8900 eligible participants (household members). The sample of this study is restricted to males, because alcohol use, as explained in the introduction, is especially pronounced among men. This study includes only male respondents over the age of 15 years. After exclusion of males under the age of 15 years, and all female in the sample, the final estimation sample of the study reduced to 2676 males. Alcohol use varies with age and young adult males are more like to consume alcohol than older groups [8, 11]. For this reason, age is categorized into four groups based on the literature [15]: 15–34 years (young adult age), 35–44 years (middle age one), 45–54 years (middle age two) and 55+ years (senior).

For alcohol use status, respondents were asked the question: does [NAME] consume alcohol? (“Often”, “Sometimes”, “Never”). A binary variable for alcohol use was constructed by setting the indicator equal to 1 if the respondent had consumed alcohol often or sometimes, and 0 for never. The dataset used in the study does not give information on when was the last time respondent consumed alcohol. In the recent study by Combes et al. [4] to investigate income related inequalities in alcohol consumption, a binary variable was constructed based on the question of whether the respondent consumed any alcohol beverage in the last 12 month. Therefore, it is assumed in this study that those who reported that they sometimes drink alcohol had consumed in the past 12 months. Further limitations associated with alcohol use variable are delineated in the discussion section.

The wealth index was used to capture the socioeconomic status of households. The socioeconomic status variable was constructed by assessing housing infrastructure and ownership of household assets. Twenty three items were taken into consideration, including the type of dwelling, source of water, toilet facilities, refuse removal source, electricity supply in the dwelling, and ownership of a refrigerator, deep freezer, domestic servant, VCR/DVD, vacuum cleaner, washing machine, computer, internet access, home service security, microwave, satellite TV, dishwasher, vehicle, iron, and electric or gas stove. The wealth index was constructed using multiple correspondence analysis (MCA). The resultant wealth index score was grouped into five quintiles, where the 1st quintile represents the poorest group and the 5th quintile represents the richest group. Other socioeconomic variables included in the study are education and employment status.

Five dummy variables of education attainment are used and they comprise of no education, primary education, secondary education, matric, and tertiary education. Employment status is described as the individual respondent’s answer of “Yes” to the question of “during the last calendar week did [NAME] work for a wage, salary, commission or any payment in kind (including paid domestic work), even if it was for only one hour?” Marital status is categorized into three groups: married or living together, widowed or divorced, and never married. Dummy variables for the nine South African provinces are included in the analysis. Race is categorized into two variables: African and Non-African (White, Indian and Coloured).

### Measuring and decomposing inequalities

To measure socioeconomic-related inequalities in health variables, Wagstaff et al. [16] and Kakwani [17] proposed the concentration index (CI). The CI can be calculated as follows

1 $$ CI=\frac{2}{\mu}\mathit{\operatorname{cov}}\left({\gamma}_i,{R}_i\right) $$

Where μ is the mean of alcohol use, y_i_ is the alcohol use of the *i*th individual and R_i_ is the cumulative percentage that the *i*th individual represent over the total population once the latter has been ranked by socioeconomic status. The concentration index is bounded between the value of negative one (− 1) and positive one (+ 1). Negative values imply that alcohol use is more concentrated among the poor population and positive values imply that alcohol use is more concentrated among the least disadvantaged population. If the CI is equal to zero it means there is no socioeconomic-related inequality in alcohol use.

Erreygers [18] proposed the corrected concentration index to account for the bounded nature of binary health variables (between zero and one). In other words, to allow for the comparison between groups of individuals that may present different levels of ill health [19]. The formula for the corrected CI is written as follows:

2 $$ E(y)=\frac{4\mu }{y^{max}-{y}^{min}} CI $$

Where μ is the mean alcohol use multiplied by four, y^max^ and y^min^ are the maximum and minimum levels of health (in our case alcohol use with values of one and zero).

According to van Doorslaer and Koolman [20], concentration indices can be decomposed using regression techniques to measure the contributions of various factors to socioeconomic related inequality of alcohol use. Since the outcome variable of this paper is a binary variable, we employ the generalized linear model (GLM) of the binomial family and with a probit link function to capture the partial effects of socioeconomic and other factors on alcohol use. This method is recommended as the suitable regression to provide consistent results for the decomposition of binary outcomes compared to ordinary probit regression, regardless of the choice of the reference category [21]. For instance, findings relating to the socioeconomic group will be consistent regardless if the poorest or least advantaged group is chosen as the reference group in the regression analysis.

This paper follows Gonzalo-Almorox and Urbanos-Garrido [19] in decomposing the socioeconomic-related inequalities in alcohol use by using Erreygers corrected concentration index:

3 $$ E=4.{\sum}_k\left({\beta}_k^m\overline{x}k\right)\ {CI}_k+{GCI}_{\varepsilon } $$

Where E is the Erreygers corrected CI, $\overline{x}$_k_ is the means of explanatory variables (socioeconomic factors and demographic factors), CI_k_ is the mean of the concentration index, ${\beta}_k^m$ are the partial effects (dy/dk) evaluated at sample means,and *GCI*_*ε*_ is the generalized concentration index for the error term. The decomposition of CI allows an estimation of the percentage contribution of socioeconomic factors to socioeconomic-related inequalities in alcohol use. Statistical analysis is performed using STATA/SE 13 and CIs were calculated using Stata’s conindex command [22].

## Results

### Descriptive statistics

Table 1 shows the descriptive statistics for males living in informal settlements. More than half of males residing in informal settlement are individuals within the age group of 15–34 years (54%).

**Table 1:** Descriptive statistics males aged 15+ (*n* = 2676)

Variable	N	Mean (95% CI)
15–34	1107	53.6 (51.5, 55.8)
35–44	426	20.6 (18.8, 22.4)
45–54	325	15.7(14.2, 17.3)
55+	207	10.0 (8.7, 11.3)
Race:		
African	2379	95.6 (94.8, 96.4)
Non-African	110	4.4 (3.6, 5.2)
Marital status:		
Married	983	42.0 (40.0, 44.0)
Widow/ divorced	84	3.6 (2.8, 4.4)
Never married	1275	54.4 (52.2, 56.5)
Educational attainment:		
No school	181	7.6 (6.5, 8.6)
Primary	610	25.4 (23.7, 27.2)
Secondary	1027	42.8 (40.8, 44.8)
Matric	468	19.5 (17.9, 21.1)
Tertiary	112	4.7 (3.8, 5.5)
Employment status:		
Employed	899	38.9 (36.9, 40.9)
Wealth status:		
Quintile 1	565	21.4 (19.9, 23.0)
Quintile 2	528	20.0 (18.5, 21.6)
Quintile 3	525	19.9 (18.4, 21.4)
Quintile 4	527	20.0 (18.4, 21.5)
Quintile 5	493	18.7 (17.2, 20.2)
Province:		
Western Cape	228	8.6 (7.6, 9.7)
Eastern Cape	232	8.8(7.7, 9.9)
Northern Cape	59	2.2 (1.7, 2.8)
Limpopo	160	6.1 (5.1, 7.0)
Mpumalanga	13	0.5 (0.2, 0.8)
Gauteng	1422	53.9 (52.0, 55.8)
North West	234	8.8 (7.8, 10.0)
Kwa-Zulu Natal	229	8.7 (7.6, 9.7)
Free State	61	2.3 (1.7, 2.9)

As expected, the majority of men living in informal settlements were Africans (96%). Only 39% of male respondents were employed. About 20% of the sample had matric and 43% had not completed secondary school

**Fig. 1:**
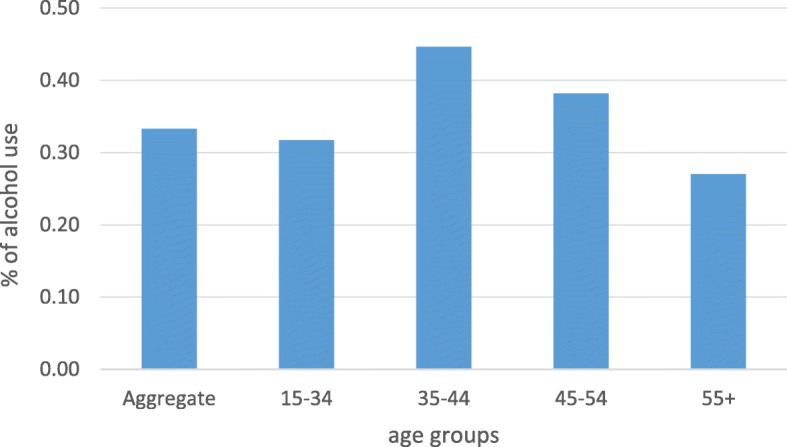
Distribution of alcohol use by males and by age group

Figure 1 shows alcohol use levels on aggregate and for each age group. About 33% of males living in informal settlements reported alcohol use and 44% of those between the age of 35 and 44 years were drinking. Alcohol use is lowest in the elderly age group.

### Inequality

Table 2 shows Erreygers concentration indices for alcohol use for separate age groups. The result shows that the concentration indices are statistically significant only for the age group 15 to 34 years and 35 to 44 years and for the aggregate, meaning that there is a socioeconomic-related inequality in alcohol use in the above-mentioned age groups.

**Table 2:** Erreygers concentration indices of alcohol use

	CI	SE	*p*-value
Overall males	− 0.061	0.022	0.006
15–34 years	− 0.116	0.034	0.001
35–44 years	− 0.104	0.056	0.065
45–55 years	0.039	0.069	0.568
55 years and above	0.014	0.038	0.715

All the concentration indices have negative signs, except for those that are not significant (45–54 years and 55 years and above). The socioeconomic inequality for the 45–54 years and 55 years and above age-group favours the well off. Inequalities in alcohol use for other age groups are concentrated among poor males. Also, the results point out that inequality is greater for males aged 15 to 34 years and aged 35 to 44 years than for other age groups. Accordingly, the subsequent analysis focuses only on the two former age groups, because the extent of socioeconomic inequality is not statistically significant for the other age groups.

### Decomposition analysis

The results of the decomposition analysis are presented in Tables 3 and 4. The columns in both tables displays elasticity, concentration index, absolute contributions, and percentage contribution for each determinant of alcohol use. Table 3 presents the results of the decomposition analysis for all males residing in informal settlements. The elasticity indicates the sensitivity of alcohol use to changes in the covariates. A positive elasticity means that the individuals with this characteristics are more likely to consume alcohol [4].

**Table 3:** Decomposition of CIs of alcohol use, males aged 15+

	Elasticity	CI	Absolute contribution	% contribution
Age:				
15–34	base	base	base	base
35–44	0.0153	−0.0172	−0.0011	1.01
45–54	− 0.0001	−0.0947	0.0000	−0.03
55-over	−0.002	0.0785	−0.0006	0.59
Total				1.57
Education:				
No education	base	base	base	base
Primary	−0.0199	−0.105	0.0084	−8.02
Secondary	−0.0386	0.0138	−0.0021	2.04
Matric	−0.0115	0.0343	−0.0016	1.52
Tertiary	−0.0054	0.2219	−0.0048	4.56
Total				0.11
Marital status:				
Married	base	base	base	base
Never married	−0.0200	0.0171	− 0.0014	1.31
Widowed/Separated	0.0032	−0.1218	−0.0015	1.49
Total				2.80
Race:				
African	base	base	base	base
Non-African	−0.0027	0.3925	−0.0042	4.02
Employment status:				
Unemployed	base	base	base	base
Employed	0.0309	−0.0266	−0.0033	3.15
Wealth status:				
Quintile 1 (Poorest)	base	base	base	base
Quintile 2	0.0132	−0.3713	−0.0195	18.76
Quintile3	0.0091	0.0280	0.001	−0.98
Quintile 4	−0.0134	0.4266	−0.0229	21.94
Quantile 5 (Richest)	−0.0218	0.8131	−0.0709	68.09
Total				107.81
Province:				
Western Cape	base	base	base	base
Gauteng	0.0283	0.0194	0.0022	−2.11
Eastern Cape	0.0134	0.1277	0.0068	−6.55
Northern Cape	0.0055	0.4892	0.0108	−10.40
Free State	0.0033	−0.1994	−0.0026	2.50
Kwa-Zulu Natal	0.0000	−0.1302	0.0000	−0.02
North West	0.0091	0.0891	0.0032	−3.10
Mpumalanga	0.0002	0.2090	0.0002	−0.17
Limpopo	−0.0032	− 0.6294	0.0081	−7.80
Total				−27.66
Residual				8.19

**Table 4:** Decomposition of CIs of alcohol use by age groups

	Males aged 15–34 years				Males aged 35–44 years			
	Elasticity	CI	Absolute contribution	% contribution	Elasticity	CI	Absolute contribution	% contribution
Education:								
No education	base	base	base	base	base	base	base	base
Primary	− 0.0693	−0.1964	0.0544	− 52.26	−0.0586	− 0.1788	0.0419	− 40.23
Secondary	0.0000	0.0057	−0.0021	2.03	−0.0661	0.0395	−0.0105	10.04
Matric	−0.0292	0.0265	−0.0031	2.97	−0.0392	0.0843	−0.0132	12.71
Tertiary	−0.009	0.2162	−0.0078	7.45	−0.0147	0.1775	−0.0105	10.05
Total				−39.82				−7.44
Marital status:								
Married	base	base	base	base	base	base	base	base
Never married	−0.0344	0.0628	−0.0086	8.29	0.0775	−0.0426	− 0.0132	12.67
Widowed/separated	0.0047	0.5094	0.0096	−9.19	0.0138	0.0881	0.0048	−4.65
Total				−0.90				8.02
Race:								
African	base	base	base	base	base	base	base	base
Non-African	−0.0059	0.3662	−0.0087	8.34	0.0022	0.4542	0.0041	−3.89
Employment status:								
Unemployed	base	base	base	base	base	base	base	base
Employed	0.0381	−0.1108	−0.0169	16.21	0.0031	0.0460	0.0006	−0.55
Wealth status:								
Quintile 1	base	base	base	base	base	base	base	base
Quintile 2	0.0113	− 0.4027	− 0.0182	17.50	0.0057	−0.3388	−0.0077	7.41
Quintile 3	0.0204	0.0238	0.0019	−1.87	−0.0226	0.0635	−0.0057	5.52
Quintile 4	−0.0238	0.4340	−0.0414	39.71	0.0013	0.4349	0.0023	−2.21
Quantile 5	− 0.0137	0.8132	−0.0444	42.65	−0.0408	0.8166	−0.1334	128.03
Total				98.00				138.75
Province								
Western Cape	base	base	base	base	base	base	base	base
Gauteng	0.0429	−0.026	−0.0045	4.29	0.1068	−0.0389	− 0.0166	15.97
Eastern Cape	0.0204	0.1372	0.0112	−10.78	0.0179	0.1552	0.0111	−10.66
Northern Cape	0.0085	0.4811	0.0164	−15.76	0.0032	0.4399	0.0057	−5.48
Free State	0.0041	−0.1633	− 0.0027	2.56	0.0043	− 0.1092	− 0.0019	1.81
Kwa-Zulu Natal	0.0057	−0.1343	−0.0031	2.95	0.0081	−0.194	−0.0063	6.03
North West	0.0144	0.1032	0.0060	−5.72	0.0052	0.1657	0.0034	−3.28
Mpumalanga	0.0013	0.2541	0.0013	−1.28	−0.0083	0.418	−0.0138	13.24
Limpopo	−0.0022	− 0.5979	0.0052	−4.96	−0.0168	− 0.5512	0.037	−35.51
Total				−28.70				−17.88
Residual				46.86				−17.01

The Erreygers concentration index was estimated for each factor related to alcohol use. A positive (negative) sign indicates that the variable has a pro-rich (pro-poor) distribution. Factors like levels of educational attainment (secondary education, matric and degree), never married, being employed and non-African, had positive concentration indices, implying that these factors were concentrated among the pro-rich population. A positive (negative) value on the absolute contribution of explanatory variables means that the inequality in alcohol use would decrease (increase) if that variable was to become equally distributed across the wealth distribution. In a straightforward way, a percentage contribution shows that an increase (decrease) in inequality of explanatory variable will increase (decrease) the degree of inequality in alcohol use.

The factors included in the decomposition analysis explained 91.81% of overall inequality in alcohol use among males. The residual term indicates that there are some unobserved heterogeneity that affects the observed socioeconomic inequality in alcohol use**.** The unexplained determinants (residuals) contributed 8.19% to alcohol use inequality.

According to the result, inequality in alcohol use in informal settlements is mainly explained by wealth status (107.81%). Factors like province have a positive absolute contribution to alcohol use inequality and this implies that inequality in alcohol use among males would decrease if the wealth was equally distributed across provinces.

Table 4 illustrates the decomposition analysis by sub-groups (males aged 15 to 34 years and males aged 35 to 44 years). The contribution of factors like race, employment status and marital status varies by age group. The contribution of employment to alcohol use inequality for the 15–34 year is significant and positive (16.22%), while for the age group of 35–44 years is negative and not significant (0.55%). The contribution of marital status for the age group of 15–34 years is negative (0.90%), while for the age group of 35–44 years the contribution is positive (8.02%). Moreover, never married status seems to have positive contribution to alcohol use inequality for both age groups. Alcohol use inequality for both age groups is mainly explained by wealth status, which accounts for 98.00% for males aged 15–34 years and 138.75% for males aged 34–45 years.

## Discussion

This paper provides evidence of the socioeconomic inequalities in alcohol use among South African males residing in informal settlements. To our knowledge, this is the first study to measure the socioeconomic gradient in alcohol use in South African informal settlements using the concentration index.

The results of the study shows that alcohol use is unequally distributed among males. Alcohol use is more concentrated among the lower socioeconomic groups. Generally, there is a little consensus in the literature on the socioeconomic gradient of alcohol use. While the socioeconomic gradient of alcohol use that is shown in this study is consistent with one previous finding [23], other studies for the most part found conflicting results that alcohol use is concentrated among pro-rich [4, 12, 13, 24]. Casswell et al. [12] investigated the relationship between several indicators of socioeconomic status and drinking patterns of young adults in New Zealand. The findings showed that the frequency of drinking was influenced by income, with higher income respondents drinking more often. Combes et al. [4] studied the income-related inequalities in alcohol use and its changes over an eight year period in Sweden. The authors found that inequality in alcohol use in Sweden is pro rich. The articles cited above used income as indicator of socioeconomic status, though in this study, a wealth index was used to measure socioeconomic status. It has been suggested in the literature that wealth index provides a reasonable proxy for measuring inequalities in the absence of income variable [25, 26]. Furthermore, the latter studies were conducted in developed countries unlike the current study, which covers only individuals living in low income areas (informal settlements). Therefore, the differences in the country’s welfare and the indicator of socioeconomic status could be the reason for the contradicting results.

The decomposition analysis undertaken in this study demonstrates that socioeconomic status (wealth status) is the main contributing factor to inequalities in alcohol use and significant in all decompositions, i.e. the total male sample as well as the two specific age groups. Thus, the results are in line with previous studies that show how socioeconomic status have an influence on alcohol use [4, 27, 28]. In addition, our results can also be explained by the large number of informal alcohol retailers in South African informal settlements [3]. According to Cain et al. [29] men who drinks in shebeens report greater quantity and frequency of alcohol used compared to those who does not patronize in shebeens. We found that the contribution of social determinants of health like employment, and marital status varies considerable by age groups. For example, employment status among males aged 15–34 is concentrated among the rich socioeconomic class and have a larger contribution to inequality of males aged 15–34 years than to older males. Stress, anxiety and depression from work were identified as causes of excessive alcohol consumption [30–32]. In addition, the contribution of marital status also differ by age group. For males aged 15–34 years the contribution is negative (0.90%) while for the age group of 35–44 years the contribution is positive (8.02%).

Never married men have a higher contribution to inequality in alcohol use for both age groups of 15–34 years and 35–44 years. This finding is consistent with a previous study [33]. Karlamangla et al. [34], specifically, found a greater risk of alcohol consumption to be associated with never married individual. This study suggests that different age groups will require different interventions to reduce inequality in alcohol use. The major strength of this study is the data used to decompose the socioeconomic inequalities in alcohol use, which was obtained from a nationally representative survey. The results are representative of informal settlements in the country. The study, moreover, explicitly focuses on an important but often neglected group, namely men living in informal settlements. The current study has some limitations that are worth considering. Firstly, the available literature on alcohol use, define persons as a current drinker if they had consumed alcohol during the past 12 months [4, 35]. The South African Study of Informal settlement used in this paper describe a person as a drinker if responded “often or sometimes” and non-drinker if responded “never” drink alcohol. Unfortunately, this is the only information provided by South African Study of Informal settlement, it does not specify whether a person is abstainer or drinker. It is possible that some respondents that drinks sometimes had not consumed alcohol in the past 12 months. In addition, information contained in the South African Study of informal Settlement does not give measures on how people consume alcohol and what type of alcohol they consume. Therefore, we acknowledge the possibility of reporting bias.

Secondly, the comparability of our findings with other studies is constrained by the fact that the studies use different measures of socioeconomic status to quantify inequalities in alcohol use. Thirdly, the residual value for the decomposition analysis of males aged 15–34 years is larger compared to other analysis. This result could be explained by a larger number of social determinants of health that are worth considering. Social determinants like parental education, parental drinking status, and stress were not included in the study. In a previous study, investigators found that the influence of stress on alcohol consumption was positive and significant [30, 36]. Fourthly, the data employed in the study is based on self-report of alcohol use which may under-report the true alcohol participation rate. Finally, cross-sectional data was used in the study, so trends in alcohol use and the associated inequalities were not investigated.

## Conclusions

This study assesses the extent of socioeconomic-related inequality in alcohol use in men living in urban informal settlements and decomposes the socioeconomic inequality of alcohol inequalities to determine how various socioeconomic factors contribute to the observed inequalities. The results shows a significant socioeconomic-related inequality in alcohol use amongst two age groups of male’s, with inequality discriminating against the poor. The study provides evidence that wealth status contribute positively to socioeconomic-related inequality in alcohol use. Factors like marital status and employment status differs by age groups. This study demonstrate the need for effective poverty reduction campaigns that will help to improve conditions that enable better health outcomes at population level as to prevent unhealthy behaviours among low socioeconomic classes [37]. Policies and interventions aimed at reducing alcohol use outlined in the 2014 WHO Global Status Report have outlined policies and interventions aimed at reducing alcohol use. These include pricing policies, marketing of alcohol beverages, drink driving policies and countermeasures [38, 39]. The socioeconomic inequality of alcohol use presented in this study can be reduced if these alcohol use reduction policies can focus on employed males aged 15–34 years and single males in both age groups to reduce alcohol use as to improve their health status.

## Abbreviations

CI: Concentration index
HSRC: Human Science Research Council
MCA: Multiple correspondence analysis
NCD: Non-communicable diseases
SANHANES: South African National Health and Nutrition Examination Survey
SES: Socio-economic status
